# The Disease Burden of *Taenia solium* Cysticercosis in Cameroon

**DOI:** 10.1371/journal.pntd.0000406

**Published:** 2009-03-31

**Authors:** Nicolas Praet, Niko Speybroeck, Rafael Manzanedo, Dirk Berkvens, Denis Nsame Nforninwe, André Zoli, Fabrice Quet, Pierre-Marie Preux, Hélène Carabin, Stanny Geerts

**Affiliations:** 1 Institute of Tropical Medicine, Antwerp, Belgium; 2 Institute of Health and Society, Université Catholique de Louvain, Brussels, Belgium; 3 Batibo District Hospital, Batibo, Cameroon; 4 University of Dschang, Dschang, Cameroon; 5 Institute of Neuroepidemiology and Tropical Neurology, Limoges, France; 6 College of Public Health, The University of Oklahoma Health Sciences Center, Oklahoma City, Oklahoma, United States of America; Universidad Peruana Cayetano Heredia, Peru

## Abstract

**Background:**

*Taenia solium* cysticercosis is an important zoonosis in many developing countries. Human neurocysticercosis is recognised as an important cause of epilepsy in regions where the parasite occurs. However, it is largely underreported and there is a lack of data about the disease burden. Because a body of information on human and porcine cysticercosis in Cameroon is becoming available, the present study was undertaken to calculate the impact of this neglected zoonosis.

**Methods:**

Both the cost and Disability Adjusted Life Year (DALY) estimations were applied. All necessary parameters were collected and imported in R software. Different distributions were used according to the type of information available for each of the parameters.

**Findings:**

Based on a prevalence of epilepsy of 3.6%, the number of people with neurocysticercosis-associated epilepsy was estimated at 50,326 (95% CR 37,299–65,924), representing 1.0% of the local population, whereas the number of pigs diagnosed with cysticercosis was estimated at 15,961 (95% CR 12,320–20,044), which corresponds to 5.6% of the local pig population. The total annual costs due to *T. solium* cysticercosis in West Cameroon were estimated at 10,255,202 Euro (95% CR 6,889,048–14,754,044), of which 4.7% were due to losses in pig husbandry and 95.3% to direct and indirect losses caused by human cysticercosis. The monetary burden per case of cysticercosis amounts to 194 Euro (95% CR 147–253). The average number of DALYs lost was 9.0 per thousand persons per year (95% CR 2.8–20.4).

**Interpretation:**

This study provides an estimation of the costs due to *T. solium* cysticercosis using country-specific parameters and including the human as well as the animal burden of the zoonotic disease. A comparison with a study in South Africa indicates that the cost of inactivity, influenced by salaries, plays a predominant role in the monetary burden of *T. solium* cysticercosis. Therefore, knowing the salary levels and the prevalence of the disease might allow a rapid indication of the total cost of *T. solium* cysticercosis in a country. Ascertaining this finding with additional studies in cysticercosis-endemic countries could eventually allow the estimation of the global disease burden of cysticercosis. The estimated number of DALYs lost due to the disease was higher than estimates already available for some other neglected tropical diseases. The total estimated cost and number of DALYs lost probably underestimate the real values because the estimations have been based on epilepsy as the only symptom of cysticercosis.

## Author Summary

*Taenia solium* cysticercosis is a zoonotic disease occurring in many developing countries. A relatively high prevalence in humans and pigs has been reported in several parts of the world, but insufficient data are available on the disease burden. Disease impact assessment needs detailed information on well-defined epidemiological and economic parameters. Our work conducted in West Cameroon over several years allowed us to collect the necessary information to estimate the impact of the parasite on the human and animal populations in this area using both cost and Disability Adjusted Life Year (DALY) estimations. This study identified the professional inactivity caused by the disease as the major loss factor in comparison to the cost of health care and losses due to infected pigs. These findings should allow a simpler estimation of the global disease burden based on information on salary levels and human cysticercosis prevalence in endemic areas of the world. In addition, the number of DALYs lost was higher than estimates already available for some other neglected tropical diseases in sub-Saharan Africa.

## Introduction

*Taenia solium* cysticercosis is an important but neglected zoonotic disease of man and pigs in many developing countries. Human cysticercosis is an under-recognised disease due to the large variety of clinical symptoms, epilepsy being recognised as the most important one, and the unavailability of appropriate diagnostic tools in endemic areas [1]. Similarly, porcine cysticercosis is under-reported due the absence of clinical symptoms in affected pigs and due to the poorly functioning meat inspection services in many endemic countries. Consequently, there is a lack of reliable data on the disease burden of cysticercosis. Although a proper assessment of the global burden of *T. solium* cysticercosis is essential, there has been so far a limited number of studies estimating the impact of this disease in endemic countries [2],[3]. The large amount of data available on porcine and human cysticercosis in the West of Cameroon (which can be considered as an endemic country) [4], allows a study of its public health and economic relevance and consequently a better estimate of the burden of *T. solium* cysticercosis which is highly needed [5]. To this end and as already achieved for other neglected diseases [6],[7], two approaches are considered namely the cost and the Disability Adjusted Life Year (DALY) estimations. A comparison with previous studies may provide indications on key factors in the more global burden of *T. solium* cysticercosis.

## Materials and Methods

### Study area

West Cameroon comprises three provinces (West, South-West and North-West) with a population of about five million inhabitants [8]. Together with the north of Cameroon the western provinces are the main pig breeding regions of the country. Pig husbandry is still very traditional. Human defecation occurs quite often in the pigsties, so that many pigs have access to human faeces [9]–[11]. All conditions are present for an easy transmission of *T. solium* from man to pigs and vice versa [9],[12]. Slaughtering of pigs usually occurs at home or on marketplaces with limited or no veterinary supervision. Tongue inspection is commonly carried out to detect cases of cysticercosis. Although there is a good network of private and public health centres in the region, the number of neurologists, neurosurgeons and psychiatrists in Cameroon is very small (8 for the whole country, nobody in West Cameroon) [13]. Imaging equipment for the diagnosis of epilepsy and neurocysticercosis (NCC) is virtually lacking. No single CT-scan or MRI is available in the whole study area. There is no public medical insurance in Cameroon and only a small number of more wealthy people have access to private insurance [14].

### Parameters for the cost estimation

The epidemiological and economic parameters used in the cost estimation are summarised in Tables 1 and 2, respectively.

**Table 1:** Epidemiological parameters used to calculate the disease burden of *T. solium* cysticercosis in West Cameroon.

ID	Parameter	Distribution	Value or range of values	Reference number
1	Population of the study zone	Fixed	5,065,382	[8]
2	Prevalence of epilepsy (%)	(1) Beta (28,1791)	(1) 1.5 (1.2–1.8)	[15],[17],[18]; see text for details
		(2) Beta (67,1791)	(2) 3.6 (2.8–4.4)	
		(3) Beta (111, 1791)	(3) 5.8 (4.8–6.9)	
3	Proportion of epilepsy associated with NCC (%)	Uniform (0.236,0.315)	27.4* (23.6–31.5)	[34]
4	Prevalence of porcine cysticercosis (%)	Beta (61,1031)	- West: 6.1	[9],[10]
			- N-West: 4.4	
5	People with epilepsy and with injury referred to the hospital (%)	Beta (58,458)	11.3	[34]
6	People with epilepsy consulting only a traditional healer (%)	Multinomial	11.1	[35]
7	People with epilepsy consulting only a medical doctor (%).	Multinomial	44.1	[35]
8	People with epilepsy consulting both a doctor and a traditional healer (%).	Multinomial	22.2	[35]
9	People with epilepsy receiving no treatment (%)	Multinomial	22.6	[35]
10	Number of visits to a doctor in case of epilepsy (per year)	Gamma (12,2)	Min.: 1	NND**
			Probable: 6	
			Max.: 12	
11	Length of stay in a hospital (days per year)	Uniform (6.5,14.5)	Min.: 7	[3]
			Max.: 14	
12	People with epilepsy prescribed pheno-barbitone (PB) (%)	Multinomial	78.7	[35]
13	People with epilepsy prescribed carbama-zepin (CBZ) (%)	Multinomial	5.0	[35]
14	People with epilepsy prescribed phenytoin (PHT) (%)	Multinomial	15.0	[35]
15	People with epilepsy prescribed valproate (%)	Multinomial	1.3	[35]
16	Degree of compliance (%)	Fixed	38.2	[35]
17	Loss of working time due to epilepsy (days per year)	Gamma (2,0.2)	10.2±18.7	[36]
18	Unemployed due to epilepsy (%)	Fixed	21.3	[35]
19	% of the population	Fixed		[8]
	- economically active		63.2	
	- not economically active		29.6	
	- unemployed		7.2	
20	Working days per year	Uniform (220,313)	Min.: 220	Assumption
			Max.: 313	
21	Pig population in the study area	Fixed	450,000	[19], Bourdanne (pers. comm.)
			121,211	
			Average: 285,606	

*: figure based on results of Ag-ELISA and Ab-ELISA. However, the latter figure was decreased by 40% because of the possible presence of transient antibodies [37].

**: author (NND: Nsame Nforninwe Denis).

**Table 2:** Economic parameters used to calculate the monetary burden of *T. solium* cysticercosis in West Cameroon.

ID	Parameter	Distribution	Value (Euro)	References
1	Cost of a visit to a physician (public hospital).	Gamma (0.5,0.5)	Min.: 0	NND*
			Probable: 1	
			Max.: 5	
2	Cost of a traditional healer	Uniform (150,180)	Min.: 150	[14]
			Max.: 180	
3	Cost of one day at the hospital	Gamma (20,2)	Min.: 5	NND*
			Probable: 10	
			Max.: 20	
4	Carbamazepin (price of treatment/day)	Fixed	0.030	PMP*
5	Phenobarbitone (price of treatment/day)	Fixed	0.0075	PMP*
6	Phenytoin sodium (price of treatment/day)	Fixed	0.0225	PMP*
7	Valproate (price of treatment/day)	Fixed	0.0225	PMP*
8	Monthly salary	Uniform (75,105)	Min.: 75	ZA*
			Max.: 105	
9	Average value of an adult pig	Fixed	100	ZA*
10	Reduction (30%) of the price of the pigs diagnosed with cysticercosis	Fixed	30	[21]

*: co-authors (NND: Nsame Nforninwe Denis; PMP: P.M. Preux; ZA: Zoli André).

**Figure 1:**
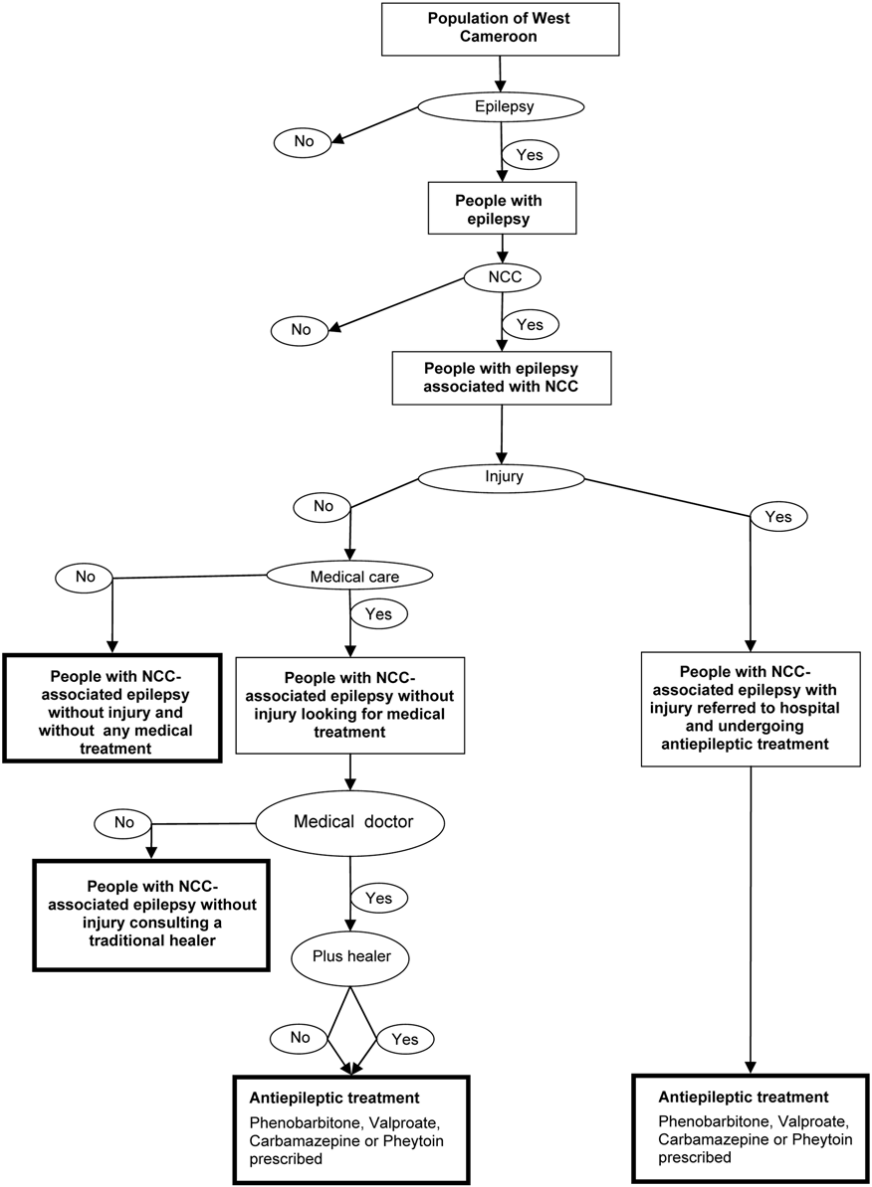
Decision tree for estimating the monetary burden of neurocysticercosis (NCC) in West Cameroon (4 terminal nodes are indicated by rectangles with bold lines).

A decision tree was constructed in order to identify the proportion of the population with epilepsy due to NCC, with or without injury, with or without treatment by a medical doctor or a traditional healer (Figure 1). Since there were no recent reliable figures available for the prevalence of epilepsy in Cameroon three scenarios were used. The first one was based on a study of Dongmo et al. (2000) [15] in which a prevalence of epilepsy of 5.8% was found based on a door-to-door survey of 1900 inhabitants of the village of Bilomo (near to Yaounde). Although this figure is probably biased due to the high level of familial epilepsy in that village, it is similar to the figure of 7.0% observed in 1989 by Nkwi&Ndonko in another small village (Maham) in West Cameroon [16]. In the second scenario we used the median prevalence of epilepsy (1.5%) found in a meta-analysis of 28 door-to-door studies in sub-Saharan Africa [17]. The third scenario used a prevalence of 3.6% (in between the extremes of 5.8 and 1.5%). These scenarios agree with epilepsy prevalence figures from Central Province of Cameroon [18].

For the estimation of the losses due to porcine cysticercosis recent prevalence figures (4.4–6.1%) based on tongue inspection were used [9],[10] because this is the only technique applied to detect the disease in pigs in the area. Since reliable information about the number of pigs was not available the pig population in West Cameroon was estimated at 285,606 based on the average of the following two figures: (1) 450,000 being one third of the FAO estimate of the total pig population of the country [19] (the other 2/3 of the pigs are present in North Cameroon) and (2) 121,211 being the number of pigs estimated by the ‘Projet Porc’ in West Cameroon (Bourdanne, pers comm., 2007). For the estimation of the pig losses it was assumed that the number of pigs slaughtered per year is the same as the total pig population. This is based on the fact that in traditional pig husbandry a similar number of pigs as the number present at the beginning of the year is slaughtered during the course of one year [20]. In Cameroon the average reduction of the price of a cysticercosis infected animal is estimated at 30% [21]. This is the only economic loss which has been taken into account because, to our knowledge, porcine cysticercosis has not been associated with clinical symptoms and consequently does not impact on productivity [22]. The following assumptions were made. The number of working days per year was estimated at minimum 220 and maximum 313 in order to take into account differences according to the economic or the informal sector. The price of a visit to a traditional healer is difficult to estimate because the healers usually ask an all-inclusive price until complete cure [14]. Assuming that there are on average 10 visits, the total price (100,000–120,000 F CFA or approximately 150 to 180 Euros) has been divided by ten.

### Parameters for the DALY estimation

The epidemiological and DALY parameters are summarised in Table 3.

**Table 3:** DALY parameters used to calculate the disease burden of *T. solium* cysticercosis in West Cameroon.

ID	Parameter	Distribution	Value	References
1	People with epilepsy dying of epilepsy per year (%)	Beta (3.2,124.8)	2.5 (0.7,5.6)	[38]
2	Epilepsy Disability Weight for people between 0 and 4 years old not receiving an appropriate treatment*	Beta (3,27.3)	0.099 (0.021,0.225)	[23]
3	Epilepsy Disability Weight for people older than 5 years old not receiving an appropriate treatment*	Beta (3,17)	0.15 (0.033,0.331)	[23]
4	Epilepsy Disability Weight for people between 0 and 4 years old receiving an appropriate treatment*	Beta (1.5,35)	0.041 (0.003,0.124)	[23]
5	Epilepsy Disability Weight for people older than 5 years old receiving an appropriate treatment*	Beta (1.5,21.6)	0.065 (0.004,0.192)	[23]
6	Average duration of disability in males between 0 and 4 years old (years)	Fixed	1.4	[23]
7	Average duration of disability in males between 5 and 14 years old (years)	Fixed	2.0	[23]
8	Average duration of disability in males between 15 and 44 years old (years)	Fixed	3.6	[23]
9	Average duration of disability in males between 45 and 59 years old (years)	Fixed	2.8	[23]
10	Average duration of disability in males older than 60 years (years)	Fixed	1.6	[23]
11	Average duration of disability in females between 0 and 4 years old (years)	Fixed	1.6	[23]
12	Average duration of disability in females between 5 and 14 years old (years)	Fixed	3.1	[23]
13	Average duration of disability in females between 15 and 44 years old (years)	Fixed	5.9	[23]
14	Average duration of disability in females between 45 and 59 years old (years)	Fixed	6.0	[23]
15	Average duration of disability in females older than 60 years (years)	Fixed	2.8	[23]

*: since no variability of these weights was available, beta distributions were arbitrarily used for each disability weight.

DALY estimation results from the sum of the number of years of life lost due to mortality (YLL) and the number of years lived with a disability (YLD) [23],[24]. YLL is calculated by accumulation over age strata of the product of the number of deaths due to the disease and the standard life expectancy at the age of death due to that disease. We have assumed that mortality affects the population in a random fashion and derive the life expectancy from the standard life table as proposed by Murray and Lopez [23]. This table is based on the highest observed national life expectancy (for Japanese women), but takes into account different life expectancy between men and women. YLD is calculated by accumulation over age strata of the product of the number of incident cases and its severity.

The formulas for the YLL and YLD calculations are described below:
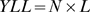
where *N* is the number of deaths per year and *L* is the standard life expectancy at age of death in year
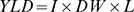
where *I* is the number of incident cases per year, *DW* is the disability weight and *L* the average duration of the disease until remission or death in year.

Three percent discounting and non-uniform age weighting were used as described by Murray and Lopez [23].

Since the annual rate of incident cases of epilepsy due to neurocysticercosis is not known in the area but expected to be low, this parameter was estimated using the prevalence of epilepsy associated with NCC described above divided by the duration of the disease for each age category [23],[25]. Therefore both incidence rate and disease duration were assumed to be constant. The three aforementioned prevalence scenarios were considered. The Global Burden of Disease 1990 disability weights for epilepsy were used in this study taking into account a difference between treated and untreated individuals [23],[26].

### Cost and DALY estimations

The R software (R Development Core Team, version 2.5.0) was used to estimate the annual socio-economic costs and number of DALYs lost due to *T. solium* cysticercosis [27],[28]. R allows to use Monte Carlo simulations and allows calculating 95% confidence regions (CR) [29]. The number of iterations was varied from 1 to 200,000 in order to achieve the optimal number. Different distributions were used according to the type of information available for each of the parameters (see Tables 1, 2 and 3 for details).

For the cost estimation, an exchange rate of 656 F CFA for 1 Euro was used.

## Results

### Cost estimation

The estimated number of people with NCC-associated epilepsy and of pigs infected with cysticercosis is shown in Table 4. From 20,000 iterations onwards the cost by NCC case did not change by more than 1%. This indicates that the results would not improve appreciably if the number of iterations would have been larger.

**Table 4:** Estimated number of people with NCC-associated epilepsy and of pigs infected with *T. solium* cysticercosis in West Cameroon.

Estimate	Number	% of total population	95% CR°
• People with NCC-associated epilepsy*	50,326**	1.0	37,299–65,924
• Pigs with cysticercosis	15,961	5.6	12,320–20,044

*: based on a prevalence of epilepsy of 3.6%. With a prevalence of 1.5 or 5.8% the numbers are 21,483 and 81,724, respectively.

**: of whom 10,076 did not receive any medical treatment.

**°:** confidence region.

The estimated direct and indirect annual costs due to *T. solium* cysticercosis in man and pigs are summarised in Table 5.

**Table 5:** Estimated direct and indirect annual costs due to *T. solium* cysticercosis in man* and pigs in West Cameroon.

Type of costs	Value (Euro)	95% CR°	% of total cost
• Hospital	595,576	312,117–1,004,400	5.8
• Medical doctor	179,001	185–968,851	1.7
• Healer	245,511	177,444–328,063	2.4
• Inactivity	8,701,883	5,600,388–12,907,812	84.9
• Drugs	54,386	40,230–71,315	0.5
• Pig losses	478,844	369,587–601,325	4.7
• **Total**	**10,255,202**	**6,889,048–14,754,044**	100.0
**Cost by NCC case** **	**194**	**147–253**	**-**

*: based on a prevalence of epilepsy of 3.6%.

**: pig losses not taken into account.

**°:** confidence region.

The economic losses due to the reduction of the value of pigs infected with cysticercosis amount to 4.7% of the total cost of the disease whereas the direct and indirect costs due to human cysticercosis (NCC) add up to 95.3%. Based on a prevalence of epilepsy of 1.5 or 5.8%, the estimated number of people with NCC-associated epilepsy is 21,483 and 81,724, respectively. In the former case the total cost is 4,649,104 whereas in the latter case it amounts to 16,331,449 Euro.

### DALY estimation

The estimated annual number of deaths due to NCC-associated epilepsy and annual number of incident cases are summarised in Table 6. The estimated annual number of DALYs lost is described in Table 7. From 20,000 iterations onwards the number of DALYs by thousand individuals did not change by more than 1%. This indicates that the results would not improve appreciably if the number of iterations would have been larger.

**Table 6:** Estimated annual number of death due to NCC-associated epilepsy and annual incident cases of epilepsy due to NCC.

Estimate	Number	95% CR°
• Annual number of deaths due to NCC-associated epilepsy*	1258	264–3073
• Annual number of incident cases of NCC-associated epilepsy**	18268	13458–23826

*: based on a prevalence of epilepsy of 3.6%. With a prevalence of 1.5 or 5.8% the numbers are 537 and 2024, respectively.

**: based on a prevalence of epilepsy of 3.6%. With a prevalence of 1.5 or 5.8% the numbers are 7779 and 29440, respectively.

**°:** confidence region.

**Table 7:** Estimated annual number of DALYs lost due to NCC-associated epilepsy in West Cameroon.

Type of DALY	Value (DALYs)	95% CR°	% of total DALYs
• YLLs*	39017.0	8195.6–95512.8	85.1
• YLDs*	6821.4	2765.1–12878.4	14.9
• **Total DALYs** **	**45838.4**	**14108.1–103469.4**	100.0
**DALYs per thousand persons** ***	**9.0**	**2.8–20.4**	**-**

*: based on a prevalence of epilepsy of 3.6%.

**: based on a prevalence of epilepsy of 3.6%. With a prevalence of 1.5 or 5.8% the numbers are 19425.4 and 73188.7, respectively.

***: based on a prevalence of epilepsy of 3.6%. With a prevalence of 1.5 or 5.8% the numbers are 3.8 and 14.4, respectively.

**°:** confidence region.

## Discussion

Carabin and colleagues were the first to make available a comprehensive estimation of the economic impact of cysticercosis [3]. These authors recognise some possible improvements, one of them being the use of context specific parameters. These were not all available in the Eastern Cape province of South Africa and some parameters had to be derived from studies conducted outside Africa. Most of the data which were used in the cost estimation of *T. solium* cysticercosis in this paper were specific for (West)-Cameroon or sub-Saharan Africa or were based on the expert opinion of some of the Cameroonian co-authors (NND, ZA). Importantly, both the animal and human burden of this zoonotic disease were included in the estimations.

Table 8 summarizes a comparison of the estimations of the disease burden due to *T. solium* cysticercosis in West Cameroon with the ones in the Eastern Cape Province of South Africa [3]. Although the number of NCC-associated cases of epilepsy is higher in Cameroon, the overall monetary burden in Cameroon (10.3 million Euro) is below the lower estimate of South Africa. The reason for this discrepancy seems to be the lower salaries in Cameroon. Indeed, inserting the salary parameters of Carabin et al. in our model resulted in very similar cost estimations in Cameroon compared to the Eastern Cape [3]. This observation together with the elevated contribution of inactivity in the total cost is an indication that the salaries in a country might be useful as an indicator of the economic impact of cysticercosis per patient. Ascertaining if knowledge on salaries and cysticercosis prevalence is sufficiently adequate to provide an initial estimation of the total cost of cysticercosis in other countries, would allow an extension towards a rapid initial estimation of the global cost of cysticercosis. Furthermore, it might be appealing to know whether or not this observation might be extendable to other chronic endemic diseases than cysticercosis.

**Table 8:** Comparison of the monetary burden of *T. solium* cysticercosis in West Cameroon and Eastern Cape Province (ECP), South Africa.

Estimate	West Cameroon (This study)	ECP, South Africa [3]
• Population	5,065,382	7,088,000
• No. (%) of NCC-associated cases of epilepsy	50,326* (1.0)	34,662 (0.5)
• Overall monetary burden (×10^6^ Euro)	10.3	15.0–27.5°
○ % due to human cysticercosis	95.3	73.1–85.4
○ % due to porcine cysticercosis	4.7	14.6–26.9
• Monetary burden per capita (Euro)	2.0	2.1–3.9

*: based on a prevalence of epilepsy of 3.6%.

**°:** different calculation methods were used (based on 2004 exchange rate of 1US$ = 0.805 Euro).

It has to be noticed that the health care system in South Africa is better organised than in Cameroon. In South Africa a relatively good working system of medical insurance exists, which totally lacks in Cameroon. This is illustrated by the total expenditure on health care per capita in Cameroon which amounts to 83 Intl $ (representing 5.2% of the Gross Domestic Product, 2004) whereas this reaches 748 Intl $ in South Africa (8.6% of the GDP, 2004) [30]. Although expensive cerebral CT scans were for instance carried out for a proportion of the South African people with NCC-associated epilepsy whereas this was not the case in Cameroon, this was not influential on the total cost differences between the two regions [3].

In addition, this paper evaluated for the fist time the burden of *T. solium* cysticercosis by estimating the number of DALYs lost due to the disease. The average annual number of DALYs lost due to *T. solium* cysticercosis in West Cameroon amounted to 9.0 per thousand persons which is about 4 times higher than the same estimations already available for trypanosomiasis and schistosomiasis in sub-Saharan Africa [31].

Our study shows some limitations. The total estimated cost of 10,255,202 Euro and the total number of DALYs lost of 45838 probably underestimate the real values because the estimations have been based on epilepsy as the only symptom of neurocysticercosis. Other symptoms like chronic headache, hydrocephalus, encephalitis, ocular cysticercosis have not been taken into account [32]. Furthermore, the stigmatisation of people with epilepsy, which seems to be very common in Africa, has also consequences which are difficult to capture. On the other hand, the proportion of epilepsy associated with NCC used is this study was based on serological results which were not confirmed using imaging and electroencephalogram. This could result in either an over- or underestimation of our results. Moreover, because estimates of the incidence of NCC-associated epilepsy were not available, the number of incident cases used in this study has been estimated using prevalence and epilepsy duration estimates. The latter are based on overall duration of epilepsy [23] underlining the lack of disease-specific information currently available, especially longitudinal epidemiological and clinical data.

Concerning porcine cysticercosis, the monetary losses were estimated using prevalence figures (4.4–6.1%) based on tongue inspection. It is known that the sensitivity of this technique is only 21% [33]. Therefore, losses due to porcine cysticercosis would be higher if better diagnostic tools would be available during meat inspection.

In conclusion, this study shows for the first time the impact of *T. solium* cysticercosis, which is a truly neglected disease in West Cameroon. The very large proportion of the overall costs attributable to inactivity as well as the uncertainty related to the other parameters calls for more extensive studies to be conducted in sub-Saharan Africa to increase the precision of these estimates. If similar studies could be carried out in a selection of other endemic countries in different parts of the world, it might be possible to understand if it is possible to extrapolate the predominant contribution of inactivity in the cysticercosis cost. This would eventually allow calculating the global disease burden for cysticercosis.

## Supporting Information

Alternative Language Abstract S1 Translation of the Abstract into French by Nicolas Praet
(0.05 MB PDF)
