# Supplementary material for: The Disease Burden of Taenia solium Cysticercosis in Cameroon
Source: PLoS Negl Trop Dis. 2009 Mar 31;3(3):e406. doi: 10.1371/journal.pntd.0000406 (PMC2656639; doi:10.1371/journal.pntd.0000406)
Supplement: Alternative Language Abstract S1 — Translation of the Abstract into French by Nicolas Praet (0.05 MB PDF) [file pntd.0000406.s001.pdf]

Introduction: La cysticercose due à *Taenia solium* est une importante zoonose dans beaucoup de pays en voie de développement. La neurocysticercose humaine est reconnue comme étant une importante cause d'épilepsie dans les zones endémiques. Cependant, celle-ci est souvent sous-rapportée et peu de données sont disponibles sur l'impact de la maladie. Basée sur la disponibilité grandissante d'information sur la cysticercose humaine et porcine au Cameroun, cette étude a été réalisée dans le but estimer l'impact de cette maladie zoonotique négligée.
Méthode: Les estimations du coût monétaire et des « Disability Adjusted Life Year » ont toutes deux été appliquées. Tous les paramètres nécessaires à cet effet ont été collectés et importés dans le logiciel R. Différentes distributions ont été utilisées selon le type d'information disponible pour chaque paramètre.
Résultats : Sur base d'une prévalence d'épilepsie de 3.6%, l'estimation du nombre de personnes atteintes d'épilepsie due à la neurocysticercose s'élève à 50,326 (95% CR 12,320-20,044) représentant 1.0% de la population locale. Le nombre de porcs détectés positifs pour la cysticercose représente, quant à lui, 5.6% de la population porcine. Le coût annuel total dû à la cysticercose à *T. solium* à l'Ouest du Cameroun a été estimé à 10,255,202 Euro (95% CR 6,889,048-14,754,044) desquels 4.7% sont dus aux pertes liées à la production de porcs et 95.3% aux pertes directes et indirectes liées à la cysticercose humaine. Le coût par cas de cysticercose humaine s'élève à 194 Euro (95% CR 147-253). Le nombre moyen de DALYs perdus s'élève à 9.0 par 1000 individus et par an (95% CR 2.8 – 20.4).
Interprétation : Cette étude fournit une estimation du coût monétaire de la cysticercose à *T. solium* utilisant des données spécifiques à la région étudiée et incluant l'impact de la maladie sur l'Homme et sur l'animal. La comparaison des résultats avec ceux issus d'une étude réalisée en Afrique du Sud indique que les coûts liés à l'inactivité professionnelle, influencés par les salaires, jouent un rôle prédominant dans le coût total. La connaissance du niveau des salaires et de la prévalence de la maladie devrait donc pouvoir permettre une estimation rapide du coût total de la maladie dans un pays donné. La confirmation de ces résultats dans d'autres zones endémiques devrait permettre une estimation de l'impact global de la maladie. Le nombre de DALYs perdus est plus élevé que la même estimation pour d'autres maladies tropicales négligées. Le coût monétaire total et le nombre total de DALYs perdus sont probablement sous-estimés car les estimations ne tiennent compte que de l'épilepsie comme symptôme de la cysticercose.
